# Fighting COVID-19 Misinformation on Social Media: Experimental Evidence for a Scalable Accuracy-Nudge Intervention

**DOI:** 10.1177/0956797620939054

**Published:** 2020-06-30

**Authors:** Gordon Pennycook, Jonathon McPhetres, Yunhao Zhang, Jackson G. Lu, David G. Rand

**Affiliations:** 1 Paul J. Hill School of Business, University of Regina; 2 Kenneth Levene Graduate School of Business, University of Regina; 3 Department of Psychology, University of Regina; 4 Sloan School of Management, Massachusetts Institute of Technology; 5 Institute for Data, Systems, and Society, Massachusetts Institute of Technology; 6 Department of Brain and Cognitive Sciences, Massachusetts Institute of Technology

**Keywords:** social media, decision making, policy making, reflectiveness, social cognition, open data, open materials, preregistered

## Abstract

Across two studies with more than 1,700 U.S. adults recruited online, we present evidence that people share false claims about COVID-19 partly because they simply fail to think sufficiently about whether or not the content is accurate when deciding what to share. In Study 1, participants were far worse at discerning between true and false content when deciding what they would share on social media relative to when they were asked directly about accuracy. Furthermore, greater cognitive reflection and science knowledge were associated with stronger discernment. In Study 2, we found that a simple accuracy reminder at the beginning of the study (i.e., judging the accuracy of a non-COVID-19-related headline) nearly tripled the level of truth discernment in participants’ subsequent sharing intentions. Our results, which mirror those found previously for political fake news, suggest that nudging people to think about accuracy is a simple way to improve choices about what to share on social media.

We’re not just fighting an epidemic; we’re fighting an infodemic.

—Tedros Adhanom Ghebreyesus (2020), Director-General of the World Health Organization

The COVID-19 pandemic represents a substantial challenge to global human well-being. Not unlike other challenges (e.g., global warming), the impact of the COVID-19 pandemic depends on the actions of individual citizens and, therefore, the quality of the information to which people are exposed. Unfortunately, however, misinformation about COVID-19 has proliferated, including on social media (Frenkel, Alba, & Zhong, 2020; Russonello, 2020).

In the case of COVID-19, this misinformation comes in many forms—from conspiracy theories about the virus being created as a biological weapon in China to claims that coconut oil kills the virus. At its worst, misinformation of this sort may cause people to turn to ineffective (and potentially harmful) remedies, as well as to either overreact (e.g., by hoarding goods) or, more dangerously, underreact (e.g., by engaging in risky behavior and inadvertently spreading the virus). As a consequence, it is important to understand why people believe and share false (and true) information related to COVID-19—and to develop interventions to increase the quality of information that people share online.

Here, we applied a cognitive-science lens to the problem of COVID-19 misinformation. In particular, we tested whether previous findings from the domain of political fake news (fabricated news stories presented as if from legitimate sources; Lazer et al., 2018) extended to misinformation related to COVID-19. We did so by drawing on a recently proposed inattention-based account of misinformation sharing on social media (Pennycook et al., 2020). According to this account, people generally wish to avoid spreading misinformation and, in fact, are often able to tell truth from falsehood; however, they nonetheless share false and misleading content because the social media context focuses their attention on factors other than accuracy (e.g., partisan alignment). As a result, they get distracted from even considering accuracy when deciding whether to share news—leading them to not implement their preference for accuracy and instead share misleading content. In support of this inattention-based account, recent findings (Pennycook et al., 2020) showed that most participants were surprisingly good at discerning between true and false political news when asked to assess “the accuracy of headlines”—yet headline veracity had very little impact on participants’ willingness to share the headlines on social media. Accordingly, subtle nudges that made the concept of accuracy salient increased the veracity of subsequently shared political content—both in survey experiments and in a large field experiment on Twitter.

It was unclear, however, how (or whether) these results would generalize to COVID-19. First, it may be that a greater level of specialized knowledge is required to correctly judge the accuracy of health information relative to political information. Thus, participants may be unable to discern truth from falsehood in the context of COVID-19, even when they do consider accuracy. Second, it was unclear whether participants would be distracted from accuracy in the way that Pennycook et al. (2020) observed for political headlines. A great deal of evidence suggests that people are motivated to seek out, believe, and share politically congenial information (Kahan, Peters, Dawson, & Slovic, 2017; Kunda, 1990; Lee, Shin, & Hong, 2018; Mercier & Sperber, 2011; Shin & Thorson, 2017). Thus, it seems likely that these partisan motivations are what distracted participants from accuracy in the study by Pennycook et al. (2020), who used highly political stimuli. If so, we would not expect similar results for COVID-19. Much of the COVID-19 information (and misinformation) circulating online is apolitical (e.g., that COVID-19 can be cured by Vitamin C). Furthermore, despite some outliers, there was (at the time these studies were run) relatively little partisan disagreement regarding the seriousness of the pandemic (Galston, 2020). Indeed, as described below, there were no partisan differences in likelihood to believe true or false COVID-19 headlines in our data. Thus, if partisanship were the key distractor, people should not be distracted from accuracy when deciding whether to share COVID-19-related content. On the contrary, one might reasonably expect the life-and-death context of COVID-19 to particularly focus attention on accuracy.

**Statement of Relevance**

Misinformation can amplify humanity’s challenges. A salient example is the COVID-19 pandemic. The environment created by the pandemic has bred a multitude of falsehoods even as truth has become a matter of life and death. In this research, we investigated why people believe and spread false (and true) news content about COVID-19. We found that people often fail to consider the accuracy of content when deciding what to share and that people who are more intuitive or less knowledgeable about science are more likely to believe and share falsehoods. We also tested an intervention to increase the truthfulness of the content shared on social media. Simply prompting people to think about the accuracy of an unrelated headline improved subsequent choices about what COVID-19 news to share. Accuracy nudges are straightforward for social media platforms to implement on top of the other approaches they are currently employing. With further optimization, interventions focused on increasing the salience of accuracy on social media could have a positive impact on countering the tide of misinformation.

In the current research, we therefore investigated the role that inattention plays in the sharing of COVID-19-related content. Study 1 tested for a dissociation between accuracy judgments and sharing intentions when participants evaluated a set of true and false news headlines about COVID-19. Study 1 also tested for correlational evidence of inattention by evaluating the relationship between truth discernment and analytic cognitive style (as well as examining science knowledge, partisanship, geographic proximity to COVID-19 diagnoses, and the tendency to overuse vs. underuse medical services). Study 2 experimentally tested whether subtly making the concept of accuracy salient increased the quality of COVID-19 information that people were willing to share online.

## Study 1

### Method

We report how we determined our sample size, all data exclusions, all manipulations, and all measures in this study. Our data, materials, and preregistration are available on the Open Science Framework (https://osf.io/7d3xh/). At the end of both surveys, we informed participants which of the headlines were accurate (by re-presenting the true headlines).

#### Participants

This study was run on March 12, 2020. We recruited 1,000 participants using Lucid, an online recruiting source that aggregates survey respondents from many respondent providers (Coppock & Mcclellan, 2019). Lucid uses quota sampling to provide a sample that is matched to the U.S. public on age, gender, ethnicity, and geographic region. We selected Lucid because it provides a sample that is reasonably representative of the U.S. population while being affordable for large samples. Our sample size was based on the following factors: (a) 1,000 is a large sample size for this design, (b) it was within our budget, and (c) it is similar to what was used in past research (Pennycook et al., 2020). In total, 1,143 participants began the study. However, 192 did not indicate using Facebook or Twitter and therefore did not complete the survey. A further 98 participants did not finish the study and were removed. The final sample consisted of 853 participants (mean age = 46 years, age range = 18–90; 357 men, 482 women, and 14 who responded “other/prefer not to answer”).

#### Materials and procedure

##### News-evaluation and news-sharing tasks

Through a partnership with Harvard Global Health Institute, we acquired a list of 15 false and 15 true news headlines relating to COVID-19 (available at https://osf.io/7d3xh/). The false headlines were deemed to be false by authoritative sources (e.g., fact-checking sites such as snopes.com and factcheck.org, health experts such as mayoclinic.com, and credible science websites such as www.livescience.com). After the study was completed, we realized that one of the false headlines (about bats being the source of the virus) was more misleading or unverified than untrue—however, removing this headline did not change our results, and so we retained it. The true headlines came from reliable mainstream media sources.

Headlines were presented in the format of Facebook posts: a picture accompanied by a headline and lede sentence. Each participant was randomly assigned to one of two conditions. In the accuracy condition, they were asked, “To the best of your knowledge, is the claim in the above headline accurate?” (yes/no). In the sharing condition, they were asked, “Would you consider sharing this story online (for example, through Facebook or Twitter?)” (yes/no); the validity of this self-report sharing measure is evidenced by the observation that news headlines that Mechanical Turk participants report a higher likelihood of sharing indeed receive more shares on Twitter (Mosleh, Pennycook, & Rand, 2020). We counterbalanced the order of the yes/no options (no/yes vs. yes/no) across participants. Headlines were presented in a random order.

A key outcome from the news task is truth *discernment*—that is, the extent to which individuals distinguish between true and false content in their judgments (Pennycook & Rand, 2019b). Discernment is defined as the difference in accuracy judgments (or sharing intentions) between true and false headlines. For example, an individual who shared 9 out of 15 true headlines and 12 out of 15 false headlines would have a discernment level of −.2 (i.e., .6 – .8), whereas an individual who shared 9 out of 15 true headlines and 3 out of 15 false headlines would have a discernment level of .4 (i.e., .6 – .2). Thus, a higher discernment score indicates a higher sensitivity to truth relative to falsity.

##### COVID-19 questions

Prior to the news-evaluation task, participants were asked two questions specific to the COVID-19 pandemic. First, they were asked, “How concerned are you about COVID-19 (the new coronavirus)?” which they answered using a sliding scale from 0 (*not concerned at all*) to 100 (*extremely concerned*). Second, they were asked “How often do you proactively check the news regarding COVID-19 (the new coronavirus)?” which they answered on a scale from 1 (*never*) to 5 (*very often*).

##### Additional correlates

We gave participants a six-item Cognitive Reflection Test (CRT; Frederick, 2005) that consisted of a reworded version of the original three-item test and three items from a nonnumeric version (we excluded the “hole” item; Thomson & Oppenheimer, 2016). The CRT is a measure of one’s propensity to reflect on intuitions (Pennycook, Cheyne, Koehler, & Fugelsang, 2016; Toplak, West, & Stanovich, 2011) and has strong test-retest reliability (Stagnaro, Pennycook, & Rand, 2018). All of the CRT items are constructed to elicit an intuitive but incorrect response. Consider, for example, the following problem: If you are running a race and pass the person in second place, what place are you in? For many people, the intuitive response of “first place” pops into mind—however, this is incorrect (if you pass the person in second place, you overtake their position and are now in second place yourself). Thus, correctly answering CRT problems is associated with reflective thinking. The CRT had acceptable reliability (Cronbach’s α = .69).

Participants also completed a general science-knowledge quiz—as a measure of general background knowledge for scientific issues—that consisted of 17 questions about basic science facts (e.g., “Antibiotics kill viruses as well as bacteria,” “Lasers work by focusing sound waves”; McPhetres & Pennycook, 2020). The scale had acceptable reliability (Cronbach’s α = .77).

We also administered the Medical Maximizer-Minimizer Scale (MMS; Scherer et al., 2016), which measures the extent to which people are either “medical maximizers” who tend to seek health care even for minor issues or, rather, “medical minimizers” who tend to avoid health care unless absolutely necessary. The MMS also had acceptable reliability (Cronbach’s α = .86).

Finally, in addition to various demographic questions, we measured political ideology on both social and fiscal issues, as well as Democrat versus Republican Party alignment.

##### Attention checks

Following the recommendations of Berinsky, Margolis, and Sances (2014), we added three screener questions that put a subtle instruction in the middle of a block of text. For example, in a block of text ostensibly about which news sources people prefer, we asked participants to select two specific options (“FoxNews.com” and “NBC.com”) to check whether they were reading the text. Full text for the screener questions, along with the full materials for the study, are available at https://osf.io/7d3xh/. Screener questions were placed just prior to the news-evaluation and news-sharing tasks, after the CRT, and after the science-knowledge scale and MMS. To maintain the representativeness of our sample, we followed our preregistered plan to include all participants in our main analyses, regardless of attentiveness. As can be seen in Table S2 in the Supplemental Material available online, our key result was robust (the effect size for the interaction between content type and condition remained consistent) across levels of attentiveness.

#### Analysis

We conducted all analyses of headline ratings at the level of the rating, using linear regression with robust standard errors clustered on participants and headline.^1^ Ratings and all individual-differences measures were *z* scored; headline veracity was coded as –0.5 for false and 0.5 for true, and condition was coded as −0.5 for accuracy and 0.5 for sharing. Our main analyses used linear probability models instead of logistic regression because the coefficients are more readily interpretable. However, logistic regression yielded qualitatively equivalent results. The coefficient on headline veracity indicates overall level of discernment (the difference between responses to true vs. false headlines), and the interaction between condition and headline veracity indicates the extent to which discernment differed between the experimental conditions.

### Results

#### Accuracy versus sharing

**Fig. 1.**
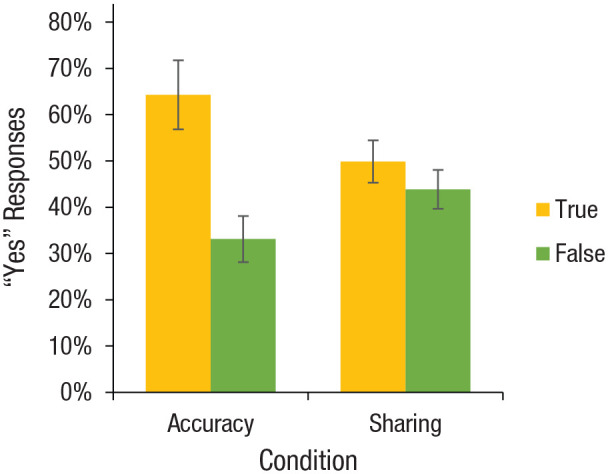
Results from Study 1: percentage of “yes” responses for each combination of headline veracity (true vs. false) and condition (accuracy = “To the best of your knowledge, is the claim in the above headline accurate?” vs. sharing = “Would you consider sharing this story online (for example, through Facebook or Twitter)?”). Error bars indicate 95% confidence intervals.

We began by comparing discernment—the difference between responses to true headlines and false headlines—across conditions. As predicted, we observed a significant interaction between headline veracity and condition, β = −0.126, *F*(1, 25586) = 42.24, *p* < .0001, indicating that discernment was higher for accuracy judgments than sharing intentions (Fig. 1; similar results were obtained when we excluded the few headlines that did not contain clear claims of fact or that were political in nature; see Table S3 in the Supplemental Material). In other words, veracity had a much bigger impact on accuracy judgments, Cohen’s *d* = 0.657, 95% confidence interval (CI) = [0.477, 0.836], *F*(1, 25586) = 42.24, *p* < .0001, than on sharing intentions, *d* = 0.121, 95% CI = [0.030, 0.212], *F*(1, 25586) = 6.74, *p* = .009. In particular, for false headlines, 32.4% more people were willing to share the headlines than rated them as accurate. In Study 2, we built on this observation to test the impact of experimentally inducing participants to think about accuracy when making sharing decisions.

#### Individual differences and truth discernment

Be-fore turning to Study 2, we examined how various individual-differences measures correlated with discernment (i.e., how individual differences interacted with headline veracity). All relationships reported below were robust to including controls for age, gender, education (college degree or higher vs. less than college degree), ethnicity (White vs. non-White), and all interactions among controls, veracity, and condition.

##### Cognitive reflection

We found that scores on the CRT were positively related to both accuracy discernment and sharing discernment, as revealed by the interactions between CRT score and veracity, *F*(1, 25582) = 34.95, *p* < .0001, and *F*(1, 25582) = 4.98, *p* = .026, respectively. However, the relationship was significantly stronger for accuracy, as indicated by the three-way interaction among CRT score, veracity, and condition, *F*(1, 25582) = 14.68, *p* = .0001. In particular, CRT score was negatively correlated with belief in false headlines and uncorrelated with belief in true headlines, whereas CRT score was negatively correlated with sharing of both types of headlines (albeit more negatively correlated with sharing of false headlines compared with true headlines; for effect sizes, see Table 1). The pattern of CRT correlations observed here for COVID-19 misinformation is therefore consistent with what has been seen previously with political headlines (Pennycook & Rand, 2019b; Ross, Rand, & Pennycook, 2019).

**Table 1.** Standardized Regression Coefficients for Simple Effects of Each Individual-Differences Measure Within Each Combination of Condition and Headline Veracity (Study 1)

Variable	Accuracy condition		Sharing condition	
	False headlines	True headlines	False headlines	True headlines
Cognitive Reflection Test score	−0.148*** (−0.127***)	0.008 (0.006)	−0.177*** (−0.174***)	−0.134*** (−0.125***)
Science knowledge	−0.080** (−0.067*)	0.079** (0.080**)	−0.082* (−0.030*)	−0.011 (−0.007)
Preference for Republican Party	0.003 (0.030)	−0.016 (−0.018)	−0.070* (−0.012)	−0.128*** (−0.079*)
Distance to nearest epicenter	−0.046† (−0.005)	−0.021 (−0.028)	−0.099** (−0.091**)	−0.099** (−0.078*)
Medical Maximizer-Minimizer Scale score	0.130*** (0.120***)	0.047* (0.051*)	0.236*** (0.0207***)	0.233*** (0.200***)

Note: Values in parentheses show the results when controls are included for age, gender, education (college degree or higher vs. less than college degree), and ethnicity (White vs. non-White) and all interactions among controls, veracity, and condition.

† *p* < .1. *p < .05. **p < .01. ***p < .001.

##### Science knowledge

Like CRT score, science knowledge was positively correlated with both accuracy discernment, *F*(1, 25552) = 32.80, *p* < .0001, and sharing discernment, *F*(1, 25552) = 10.02, *p* = .002, but much more so for accuracy, as revealed by the three-way interaction among science knowledge, veracity, and condition, *F*(1, 25552) = 7.59, *p* = .006. In particular, science knowledge was negatively correlated with belief in false headlines and positively correlated with belief in true headlines, whereas science knowledge was negatively correlated with sharing of false headlines and uncorrelated with sharing of true headlines (for effect sizes, see Table 1).

##### Exploratory measures

Distance from the nearest COVID-19 epicenter (defined as a county with at least 10 confirmed coronavirus cases when the study was run; log-transformed because of right skew) was not signifi-cantly related to belief in either true or false headlines but was negatively correlated with sharing intentions for both true and false headlines—no significant interactions with veracity, *p* > .15; the interaction between distance and condition was marginal, *F*(1, 25522) = 3.07, *p* = .080. MMS score was negatively correlated with accuracy discernment, *F*(1, 25582) = 11.26, *p* = .0008. Medical maximizers showed greater belief in both true and false headlines (this pattern was more strongly positive for belief in false headlines); in contrast, there was no such correlation with sharing discernment, *F*(1, 25582) = 0.03, *p* = .87. Thus, medical maximizers were more likely to consider sharing both true and false headlines to the same degree, as revealed by the significant three-way interaction among maximizer-minimizer, veracity, and condition, *F*(1, 25582) = 7.58, *p* = .006. Preference for the Republican Party over the Democratic Party (partisanship) was not significantly related to accuracy discernment, *F*(1, 25402) = 0.45, *p* = .50, but was significantly negatively related to sharing discernment, *F*(1, 25402) = 8.28, *p* = .004. Specifically, stronger Republicans were less likely to share both true and false headlines but were particularly less likely (relative to Democrats) to share true headlines—however, the three-way interaction among partisanship, veracity, and condition was not significant, *F*(1, 25402) = 1.62, *p* = .20. For effect sizes, see Table 1.

#### Individual differences and COVID-19 attitudes

Finally, in Table 2, we report an exploratory analysis of how all of the above variables relate to concern about COVID-19 and how often people proactively check COVID-19-related news (self-reported). Both measures were negatively correlated with CRT score and preference for the Republican Party over the Democratic Party, positively correlated with being a medical maximizer, and unrelated to science knowledge when we used pairwise correlations but significantly positively related to science knowledge in models with all covariates plus demographic controls. Distance to the nearest county with at least 10 COVID-19 diagnoses was uncorrelated with concern and negatively correlated with news checking (although uncorrelated with news checking in the model with all measures and controls).

**Table 2.** Pairwise Correlations Among Concern About COVID-19, Proactively Checking News About COVID-19, and the Individual-Differences Measures (Study 1)

Variable	COVID-19 concern	COVID-19 news checking	CRT score	Science knowledge	Partisanship (Republican)	Distance to nearest epicenter
COVID-19 concern	—					
COVID-19 news checking	.64***	—				
Cognitive Reflection Test (CRT) score	−.22*** (−0.17***)	−.10* (−0.07*)	—			
Science knowledge	−.001 (0.10**)	.06 (0.10**)	.40***	—		
Partisanship (Republican)	−.27*** (−0.19***)	−.21*** (−0.15***)	.09**	−.08*	—	
Distance to nearest epicenter	−.05 (−0.02)	−.07* (−0.04)	.01	−.03	.10*	—
Medical maximizing	.41*** (0.36***)	.36*** (0.34***)	−.23***	−.16***	−.15***	−.05

Note: Values in parentheses are standardized coefficients from linear regression models including all individual-differences measures as well as age, gender, education (college degree or higher vs. less than college degree), and ethnicity (White vs. non-White).

* *p* < .05. **p < .01. ***p < .001.

## Study 2

Study 1 established that people do not seem to readily consider accuracy when deciding what to share on social media. In Study 2, we tested an intervention in which participants were subtly induced to consider accuracy when making sharing decisions.

### Method

#### Participants

This study was run from March 13 to March 15, 2020. Following the same sample-size considerations as in Study 1, we recruited 1,000 participants using Lucid. In total, 1,145 participants began the study. However, 177 did not indicate using Facebook or Twitter and therefore did not complete the survey. A further 112 participants did not complete the study. The final sample consisted of 856 participants (mean age = 47 years, age range = 18–86; 385 men, 463 women, and 8 who responded “other/prefer not to answer”).

#### Materials and procedure

##### Accuracy induction

Each participant was randomly assigned to one of two conditions. In the control condition, they began the news-sharing task as in Study 1. In the treatment condition, they rated the accuracy of a single headline (unrelated to COVID-19) before beginning the news-sharing task; following Pennycook et al. (2020), we framed this as being for a pretest. Each participant saw one of four possible headlines, all politically neutral and unrelated to COVID-19 (see https://osf.io/7d3xh/ for materials). An advantage of this design is that the manipulation is subtle and not explicitly linked to the main task. Thus, it is unlikely that any between-conditions difference was driven by participants’ believing that the accuracy question at the beginning of the treatment condition was designed to make them take accuracy into account when making sharing decisions during the main experiment. It is therefore relatively unlikely that any treatment effect was due to demand characteristics or social desirability.

##### News-sharing task

Participants were shown the same headlines as for Study 1 and (as in the sharing condition of Study 1) were asked about their willingness to share the headlines on social media. In this case, however, we sought to increase the sensitivity of the measure by asking, “If you were to see the above on social media, how likely would you be to share it?” which they answered on a 6-point scale from 1 (*extremely unlikely*) to 6 (*extremely likely*). As described above, some evidence in support of the validity of this self-report sharing-intentions measure comes from Mosleh, Pennycook, and Rand (2020). Further support for the specific paradigm used in this study—in which participants are asked to rate the accuracy of a headline and then go on to indicate sharing intentions—comes from Pennycook et al. (2020), who found similar results using this paradigm on Mechanical Turk and Lucid and in a field experiment on Twitter measuring actual (rather than hypothetical) sharing.

##### Other measures

All of the additional measures included in Study 1 were also included in Study 2.

##### Attention checks

The same screener questions included in Study 1 were also included in Study 2. As in Study 1, to maintain the sample’s representativeness, we present the results for all participants in the main text and show the robustness of our key result across levels of attentiveness in the Supplemental Material (see Table S5).

#### Analysis

All analyses were conducted at the level of the rating, using linear regression with robust standard errors clustered on participants and headline. Sharing intentions were rescaled such that 1 on the 6-point Likert scale was 0, and 6 on the 6-point Likert scale was 1.

### Results

**Fig. 2.**
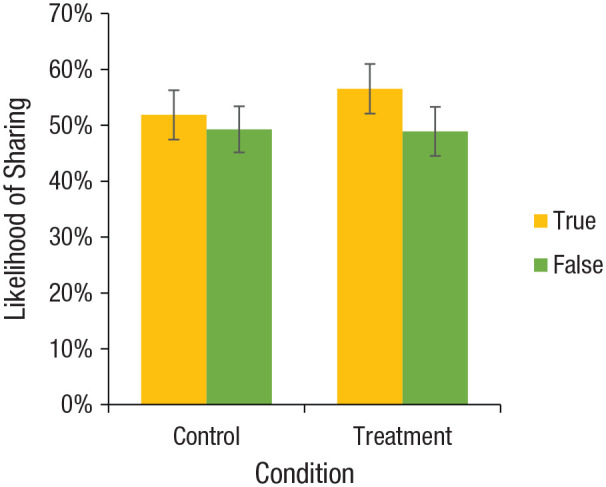
Results from Study 2: percentage of headlines participants said they would be likely to share, separately for each combination of headline veracity (true vs. false) and condition (control vs. treatment). For this visualization, we discretize sharing intentions using the scale midpoint (i.e., 1–3 = 0, 4–6 = 1) to give a more easily interpretable measurement; all analyses are conducted using the full (nondiscretized) scale, and plotting the average (nondiscretized) sharing intentions looks qualitatively similar. For the equivalent plot using mean sharing intentions instead of the discretized percentages, see Figure S1 in the Supplemental Material available online. Error bars indicate 95% confidence intervals.

As predicted, we observed a significant positive interaction between headline veracity and treatment, β = 0.039, *F*(1, 25623) = 17.88, *p* < .0001; the treatment increased sharing discernment (i.e., participants were more likely to share true headlines relative to false headlines after they rated the accuracy of a single non-COVID-related headline; Fig. 2). Specifically, although participants in the control condition were not significantly more likely to say that they would share true headlines compared with false headlines, *d* = 0.050, 95% CI = [−0.033, 0.133], *F*(1, 25623) = 1.41, *p* = .24, in the treatment condition, sharing intentions for true headlines were significantly higher than for false headlines, *d* = 0.142, 95% CI = [0.049, 0.235], *F*(1, 25623) = 8.89, *p* = .003. Quantitatively, sharing discernment (the difference in sharing likelihood of true relative to false headlines) was 2.8 times higher in the treatment condition compared with the control condition. Furthermore, the treatment effect on sharing discernment was not significantly moderated by CRT performance, science knowledge, partisanship, distance to the nearest epicenter, or MMS score (*p*s >.10 for all three-way interactions among headline veracity, treatment, and individual-differences measure). The treatment effect was also robust to excluding the few headlines that did not contain clear claims of fact or that were political in nature (see Table S6 in the Supplemental Material).

**Fig. 3.**
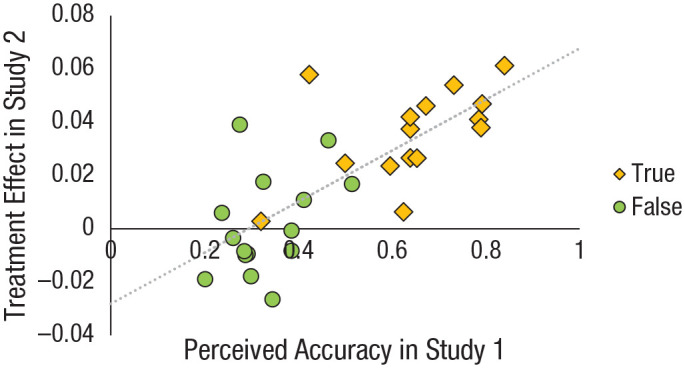
Relationship between the effect of the treatment in Study 2 and the average accuracy rating from participants in the accuracy condition of Study 1 as a function of headline veracity (true vs. false). The dashed line shows the best-fitting regression.

Our interpretation of the treatment effect is that the accuracy nudge makes participants more likely to consider accuracy when deciding whether to share. Given this mechanism, the extent to which the treatment increases or decreases sharing of a given headline should reflect the underlying perceptions of the headline’s accuracy. That is, increasing an individual’s attention to accuracy should yield the largest changes in sharing intentions for headlines that are more unilaterally perceived to be true or false. To provide evidence for such a relationship, we performed a post hoc item-level analysis. For each headline, we examined how the effect of the treatment on sharing (i.e., average sharing intention in the treatment condition minus average sharing intention in the control condition) varied on the basis of the average accuracy rating given to that headline by participants in the accuracy condition of Study 1. Because participants in Study 2 did not rate the accuracy of the COVID-19-related headlines, we used average Study 1 ratings as a proxy for how accurate participants in Study 2 would likely deem the headlines to be. As shown in Figure 3, there was indeed a strong positive correlation between a headline’s perceived accuracy and the impact of the treatment, *r*(28) = .76, *p* < .0001. Headlines that were more likely to be identified as true (on the basis of Study 1 data) were more strongly positively impacted (sharing increases) by nudging people to consider accuracy. This suggests that the accuracy nudge, as we hypothesized, increased people’s attention to whether the headlines seem true or not when they decided what to share.

## Discussion

Our results are consistent with an inattention-based account (Pennycook et al., 2020) of COVID-19-misinformation transmission on social media. In Study 1, participants were willing to share fake news about COVID-19 that they would have apparently been able to identify as being untrue if they were asked directly about accuracy. Put differently, participants were far less discerning if they were asked about whether they would share a headline on social media than if they were asked about its accuracy. Furthermore, individuals who were more likely to rely on their intuitions and who were lower in basic scientific knowledge were worse at discerning between true and false content (in terms of both accuracy and sharing decisions). In Study 2, we demonstrated the promise of a behavioral intervention informed by this inattention-based account. Prior to deciding which headlines they would share on social media, participants were subtly primed to think about accuracy by being asked to rate the accuracy of a single non-COVID-related news headline. This minimal, content-neutral intervention nearly tripled participants’ level of discernment between sharing true and sharing false headlines.

This research has important theoretical and practical implications. Theoretically, our findings shed new light on the perspective that inattention plays an important role in the sharing of misinformation online. By demonstrating the role of inattention in the context of COVID-19 misinformation (rather than politics), our results suggest that partisanship is not, apparently, the key factor distracting people from considering accuracy on social media. Instead, the tendency to be distracted from accuracy on social media seems more general. Thus, it seems likely that people are being distracted from accuracy by more fundamental aspects of the social media context. For example, social media platforms provide immediate, quantified feedback on the level of approval from one’s social connections (e.g., “likes” on Facebook). Thus, attention may by default be focused on other factors, such as concerns about social validation and reinforcement (e.g., Brady, Crockett, & Van Bavel, 2020; Crockett, 2017) rather than accuracy. Another possibility is that because news content is intermixed with content in which accuracy is not relevant (e.g., baby photos, animal videos), people may habituate to a lower level of accuracy consideration when in the social media context. The finding that people are inattentive to accuracy even when making judgments about sharing content related to a global pandemic raises important questions about the nature of the social media ecosystem.

The present studies also add to the literature on reasoning and truth discernment. While much of the discussion around fake news has focused on political ideology and partisan identity (Beck, 2017; Kahan, 2017; Taub, 2017; Van Bavel & Pereira, 2018), our data are more consistent with recent studies on political misinformation that provide both correlational (Pennycook & Rand, 2019b; including data from Twitter sharing, Mosleh, Pennycook, Arechar, & Rand, 2020) and experimental (Bago, Rand, & Pennycook, 2020) evidence for an important role of analytic cognitive style. That is, our data suggest that an important contributor to lack of truth discernment for health misinformation is the type of intuitive or emotional thinking that has been associated with conspiratorial beliefs (Swami, Voracek, Stieger, Tran, & Furnham, 2014; Vitriol & Marsh, 2018) and superstition (Elk, 2013; Lindeman & Svedholm, 2012; Risen, 2016). These findings highlight the importance of reflecting on incorrect intuitions and avoiding the traps of cognitive miserliness for a variety of psychological outcomes and regardless of political ideology (Pennycook, Fugelsang, & Koehler, 2015; Stanovich, 2005).

From a practical perspective, misinformation is a particularly significant problem in uncertain news environments (e.g., immediately following a major news event; Starbird, 2019; Starbird, Maddock, Orand, Achterman, & Mason, 2014). In cases where having high quality information may literally be a matter of life and death—such as for COVID-19—the need to develop interventions to fight misinformation becomes even more crucial. Consistent with recent work on political misinformation (Fazio, 2020; Pennycook et al., 2020), the present results show that simple and subtle reminders about the concept of accuracy may be sufficient to improve people’s sharing decisions regarding information about COVID-19 and therefore improve the accuracy of the information about COVID-19 on social media. Although accuracy nudges are far from a complete solution, the intervention may nonetheless have important downstream effects on the overall quality of information shared online (e.g., because of network effects; see Pennycook et al., 2020). Furthermore, our treatment translates directly into a suite of real-world interventions that social media companies could easily deploy by periodically asking users to rate the accuracy of randomly sampled headlines. Such ratings could also potentially help identify misinformation via crowdsourcing (Pennycook & Rand, 2019a)—especially given that, at least for the 30 headlines considered here, participants (on average) rated the true headlines as much more accurate than the false headlines.

Our research has several limitations. First, our evidence is restricted to the United States and therefore needs to be tested elsewhere in the world. Next, although our sample was quota matched to the U.S. population on age, gender, ethnicity, and region, it was not obtained via probability sampling and therefore should not be considered truly nationally representative. We also used a particular set of true and false headlines about COVID-19. It is important for future work to test the generalizability of our findings to other headlines and to information (and misinformation) about COVID-19 that comes in forms other than headlines (e.g., e-mails, text posts, and memes about supposed disease cures). Finally, our sharing intentions were hypothetical, and our experimental accuracy induction was performed in a “lab” context. Thus, one may be concerned about whether our results will extend to naturalistic social media contexts. As mentioned above, we see three reasons to expect that our results will generalize to real sharing behavior. First, there is evidence (at the level of the headline) that self-reported sharing intentions correlate meaningfully with actual sharing on social media platforms (Mosleh, Pennycook, & Rand, 2020). Second, because our manipulation was quite subtle, we believe it is unlikely that differences in sharing intentions between the treatment and control conditions (as opposed to overall sharing levels) are driven by demand effects or social desirability bias. Third, past research using similar methods has shown evidence of external validity: Pennycook et al. (2020) targeted the same accuracy-reminder intervention at political misinformation and found that the results from the survey experiments were replicated when they delivered the intervention via direct message on Twitter, significantly improving the quality of subsequent tweets from individuals who are prone to sharing misleading political news content.

## Conclusion

Our results shed light on why people believe and share misinformation related to COVID-19 and point to a suite of interventions based on accuracy nudges that social media platforms could directly implement. Such interventions are easily scalable and do not require platforms to make decision about what content to censor. We hope that social media platforms will consider this approach in their efforts to improve the quality of information shared online.

## Supplemental Material

Pennycook_OpenPracticesDisclosure_rev – Supplemental material for Fighting COVID-19 Misinformation on Social Media: Experimental Evidence for a Scalable Accuracy-Nudge Intervention Click here for additional data file. Supplemental material, Pennycook_OpenPracticesDisclosure_rev for Fighting COVID-19 Misinformation on Social Media: Experimental Evidence for a Scalable Accuracy-Nudge Intervention by Gordon Pennycook, Jonathon McPhetres, Yunhao Zhang, Jackson G. Lu and David G. Rand in Psychological Science

Pennycook_Supplemental_Material_rev – Supplemental material for Fighting COVID-19 Misinformation on Social Media: Experimental Evidence for a Scalable Accuracy-Nudge Intervention Click here for additional data file. Supplemental material, Pennycook_Supplemental_Material_rev for Fighting COVID-19 Misinformation on Social Media: Experimental Evidence for a Scalable Accuracy-Nudge Intervention by Gordon Pennycook, Jonathon McPhetres, Yunhao Zhang, Jackson G. Lu and David G. Rand in Psychological Science

## Transparency

*Action Editor:* Marc J. Buehner

*Editor:* Patricia J. Bauer

*Author Contributions*

G. Pennycook and D. G. Rand developed the study concept. J. McPhetres created the survey. All authors designed the study. G. Pennycook, J. McPhetres, and D. G. Rand analyzed the data. All authors interpreted the data. G. Pennycook and D. G. Rand drafted the manuscript, and all authors provided critical revisions. All authors approved the final version of the manuscript for submission.

**Open Practices:** All data and materials have been made publicly available via the Open Science Framework and can be accessed at https://osf.io/7d3xh/. The design and analysis plans for Studies 1 and 2 were preregistered at AsPredicted (copies of the preregistration can be seen at https://osf.io/7d3xh/). Deviations from the preregistration are noted in the text. The complete Open Practices Disclosure for this article can be found at http://journals.sagepub.com/doi/suppl/10.1177/0956797620939054. This article has received the badges for Open Data, Open Materials, and Preregistration. More information about the Open Practices badges can be found at http://www.psychologicalscience.org/publications/badges.

## Notes

1. Our preregistration erroneously indicated that we would cluster standard errors only on participant; doing so does not qualitatively change the results.
