# Supplementary material for: Fighting COVID-19 Misinformation on Social Media: Experimental Evidence for a Scalable Accuracy-Nudge Intervention
Source: Psychol Sci. 2020 Jun 30;31(7):770–80. doi: 10.1177/0956797620939054 (PMC7366427; doi:10.1177/0956797620939054)
Supplement: Pennycook_Supplemental_Material_rev – Supplemental material for Fighting COVID-19 Misinformation on Social Media: Experimental Evidence for a Scalable Accuracy-Nudge Intervention [file Pennycook_Supplemental_Material_rev.docx]

**Supplementary Materials**

***Table S1.*** *Study 1 Item Analysis*

|  |  | **All** | | **Democrats** | | **Republicans** | |
| --- | --- | --- | --- | --- | --- | --- | --- |
| **Headline** | **Item Name** | **%Accurate** | **%Shared** | **%Accurate** | **%Shared** | **%Accurate** | **%Shared** |
| $425M in World Bank catastrophe bonds set to default | False-1 | 0.38 | 0.38 | 0.4 | 0.44 | 0.35 | 0.3 |
| Is colloidal silver a cure for the coronavirus? | False-2 | 0.24 | 0.39 | 0.22 | 0.44 | 0.26 | 0.33 |
| Coronavirus: North Korea's first confirmed patient shot dead | False-3 | 0.26 | 0.39 | 0.25 | 0.41 | 0.27 | 0.36 |
| Coconut oil's history in destroying viruses, including coronaviruses | False-4 | 0.2 | 0.45 | 0.22 | 0.46 | 0.17 | 0.44 |
| Governor Cuomo signs law using coronavirus as an excuse to take 'temporary' dictator powers | False-5 | 0.3 | 0.32 | 0.28 | 0.35 | 0.32 | 0.28 |
| 328 Chinese nationals caught entering US illegally | False-6 | 0.34 | 0.42 | 0.32 | 0.43 | 0.38 | 0.4 |
| Vatican confirms Pope Francis and two aides test positive for coronavirus | False-7 | 0.28 | 0.44 | 0.27 | 0.49 | 0.27 | 0.38 |
| Florida hospital reports a coronavirus infestation with multiple confirmed patients | False-8 | 0.46 | 0.48 | 0.5 | 0.49 | 0.42 | 0.47 |
| Coronavirus in China: 23M quarantined, 2.8M infected, 112,000 dead | False-9 | 0.41 | 0.48 | 0.4 | 0.54 | 0.43 | 0.41 |
| Vitamin C protects against Coronavirus | False-10 | 0.29 | 0.47 | 0.29 | 0.46 | 0.3 | 0.47 |
| University of Tennessee scientists may have found cure | False-11 | 0.29 | 0.58 | 0.27 | 0.6 | 0.31 | 0.56 |
| Unbelievable- Gates Foundation predicted 65 million death 3 months ago | False-12 | 0.39 | 0.48 | 0.38 | 0.53 | 0.39 | 0.42 |
| FEMA proposes martial law to contain virus \| New World Order | False-13 | 0.28 | 0.42 | 0.29 | 0.46 | 0.27 | 0.37 |
| COVID-19 is now mutating into something indescribable | False-14 | 0.33 | 0.4 | 0.35 | 0.45 | 0.3 | 0.34 |
| Experts think bats are the source of the Wuhan Coronavirus. At least 4 pandemics have originated in these animals | False-15 | 0.51 | 0.43 | 0.51 | 0.48 | 0.53 | 0.38 |
| Spread of virus appears inevitable in US | True-1 | 0.79 | 0.55 | 0.81 | 0.64 | 0.76 | 0.43 |
| Trump spent the past 2 years slashing the government agencies responsible for handling the coronavirus outbreak | True-2 | 0.42 | 0.46 | 0.54 | 0.58 | 0.27 | 0.31 |
| Coronavirus infections increase in Italy | True-3 | 0.84 | 0.54 | 0.82 | 0.6 | 0.86 | 0.47 |
| Why airport screening won't stop the spread of the coronavirus | True-4 | 0.64 | 0.49 | 0.67 | 0.53 | 0.6 | 0.44 |
| Europe's outbreak worsens; Italy at forefront | True-5 | 0.78 | 0.53 | 0.78 | 0.6 | 0.8 | 0.45 |
| Coronavirus: Many people in US will be exposed at some point | True-6 | 0.79 | 0.57 | 0.78 | 0.64 | 0.8 | 0.5 |
| CDC: coronavirus spread may last into 2021; impact can be blunted | True-7 | 0.62 | 0.49 | 0.64 | 0.55 | 0.6 | 0.41 |
| Israel declared 14-day quarantine for all arrivals | True-8 | 0.67 | 0.54 | 0.66 | 0.57 | 0.69 | 0.51 |
| coronavirus poses tough challenge for economic policymakers | True-9 | 0.79 | 0.48 | 0.77 | 0.55 | 0.81 | 0.38 |
| scientists warn nCoV more infectious than SARS, experts have doubts | True-10 | 0.6 | 0.41 | 0.6 | 0.47 | 0.6 | 0.33 |
| Coronavirus: we need to start preparing for the next viral outbreak now | True-11 | 0.73 | 0.51 | 0.77 | 0.57 | 0.68 | 0.43 |
| Amazon plans to prosecute sellers for price gouging during outbreak | True-12 | 0.64 | 0.61 | 0.63 | 0.64 | 0.65 | 0.58 |
| Amid outbreak, Carnival Cruise Line offers on -ship credits to passengers who don't reschedule | True-13 | 0.65 | 0.46 | 0.65 | 0.46 | 0.65 | 0.45 |
| Iran now has the highest coronavirus death toll outside of China, threatening wider middle east | True-14 | 0.5 | 0.48 | 0.48 | 0.54 | 0.52 | 0.41 |
| Police in the US spread a false claim that meth is contaminated with virus | True-15 | 0.32 | 0.35 | 0.33 | 0.41 | 0.31 | 0.27 |

***Table S2.*** *Study 1 main analysis for each attentiveness threshold. Unlike the main text analysis, here ratings (the dependent variable) are not z-scored so that outcome values are consistent across attentiveness levels.*

|  | (1) | (2) | (3) | (4) | |
| --- | --- | --- | --- | --- | --- |
|  | All Subjects | ≥1 Screener Correct | ≥2 Screener Correct | All 3 Screeners Correct | |
|  |  |  |  |  | |
| Veracity (F=-0.5, T=0.5) | 0.186*** | 0.195*** | 0.214*** | 0.269*** | |
|  | (0.0289) | (0.0300) | (0.0318) | (0.0426) | |
| Condition (Acc=-0.5, Sharing=0.5) | -0.0185 | -0.0254 | -0.0213 | -0.0166 | |
|  | (0.0261) | (0.0265) | (0.0283) | (0.0406) | |
| Veracity X Condition | -0.252*** | -0.266*** | -0.297*** | -0.325*** | |
|  | (0.0387) | (0.0399) | (0.0421) | (0.0548) | |
| Constant | 0.478*** | 0.462*** | 0.441*** | 0.420*** | |
|  | (0.0169) | (0.0174) | (0.0185) | (0.0260) | |
|  |  |  |  |  | |
| Observations | 25,590 | 24,210 | 18,630 | 5,730 | |
| Subject clusters | 853 | 807 | 621 | 191 | |
| Headline clusters | 30 | 30 | 30 | 30 | |
| R-squared | 0.050 | 0.056 | 0.067 | 0.101 | |
| Standard errors in parentheses | | | | |  |
| *** p<0.001, ** p<0.01, * p<0.05 | | | | |  |

***Table S3.*** *Study 1 main analysis robustness checks when excluding various headlines. Model 2 excludes headlines False-2 (which was phrased a question), False-11 (which used hedged language and so was not strictly false), and Real-9 and Real-11 (which were opinion statements). Model 3 excludes headlines False-5, False-13, and True-2, which are explicitly political in nature. Model 4 excludes those three headlines plus False-12, True-1, True-9, True-11, and True-15, which are somewhat related to politics and policy. Models 5-7 excludes headlines based on the level of partisan disagreement observed in accuracy ratings from Study 1 (defined as the absolute value of the difference between average accuracy rating given by Democrats and average accuracy rating given by Republicans).*

|  | (1) | (2) | (3) | (4) | (5) | (6) | (7) |
| --- | --- | --- | --- | --- | --- | --- | --- |
|  | All headlines | Only claim of fact | No political (narrow) | No political (broad) | Dem-Rep diff < 0.1 | Dem-Rep diff < 0.05 | Dem-Rep diff < 0.025 |
|  |  |  |  |  |  |  |  |
| Veracity (F=-0.5, T=0.5) | 0.372*** | 0.351*** | 0.374*** | 0.405*** | 0.391*** | 0.388*** | 0.399*** |
|  | (0.0578) | (0.0637) | (0.0583) | (0.0552) | (0.0576) | (0.0667) | (0.0606) |
| Condition (Acc=-0.5, Sharing=0.5) | -0.0370 | -0.0357 | -0.0449 | -0.0493 | -0.0498 | -0.0433 | -0.0468 |
|  | (0.0522) | (0.0521) | (0.0530) | (0.0539) | (0.0517) | (0.0560) | (0.0550) |
| Veracity X Condition | -0.504*** | -0.431*** | -0.539*** | -0.554*** | -0.529*** | -0.522*** | -0.460*** |
|  | (0.0775) | (0.0767) | (0.0795) | (0.0819) | (0.0761) | (0.0874) | (0.0841) |
| Constant | 0.000369 | -0.00646 | 0.0181 | 0.0273 | 0.00968 | 0.00748 | 0.0376 |
|  | (0.0338) | (0.0364) | (0.0340) | (0.0327) | (0.0337) | (0.0376) | (0.0351) |
|  |  |  |  |  |  |  |  |
| Observations | 25,590 | 22,178 | 23,031 | 18,766 | 24,737 | 19,619 | 11,942 |
| Subject clusters | 853 | 853 | 853 | 853 | 853 | 853 | 853 |
| Headline clusters | 30 | 26 | 27 | 22 | 29 | 23 | 14 |
| R-squared | 0.050 | 0.042 | 0.053 | 0.059 | 0.055 | 0.054 | 0.051 |
| Standard errors in parentheses | | | | | | | |
| *** p<0.001, ** p<0.01, * p<0.05 | | | | | | | |

***Table S4.*** *Study 1 main analysis performed using multi-level model with maximal crossed random effects for subject and headline.*

Scaled residuals:

Min 1Q Median 3Q Max

-2.98953 -0.65669 -0.02514 0.71164 2.81421

Random effects:

Groups Name Variance Std.Dev. Corr

Subject (Intercept) 0.30109 0.5487

Veracity 0.14390 0.3793 -0.12

Headline (Intercept) 0.02438 0.1562

Condition 0.04045 0.2011 -0.64

Residual 0.58202 0.7629

Number of obs: 25590, groups: id, 853; item_num, 30

Fixed effects:

Estimate Std. Error df t value Pr(>|t|)

(Intercept) 0.000369 0.034477 56.314044 0.011 0.991

Veracity 0.372246 0.059253 30.904457 6.282 5.58e-07 ***

Condition -0.037028 0.053401 106.157179 -0.693 0.490

Veracity:Condition -0.503949 0.080200 34.928044 -6.284 3.30e-07 ***

---

Signif. codes: 0 ‘***’ 0.001 ‘**’ 0.01 ‘*’ 0.05 ‘.’ 0.1 ‘ ’ 1

Correlation of Fixed Effects:

(Intr) Veracity Sharing

Veracity -0.014

Condition -0.371 0.000

Verac:Cond 0.000 -0.565 -0.027

***Table S5.*** *Study 2 main analysis for each attentiveness threshold.*

|  | (1) | (2) | (3) | (4) |
| --- | --- | --- | --- | --- |
|  | All Subjects | ≥1 Screener Correct | ≥2 Screener Correct | All 3 Screeners Correct |
|  |  |  |  |  |
| Veracity (F=0, T=1) | 0.0191 | 0.0218 | 0.0288 | 0.0297 |
|  | (0.0161) | (0.0164) | (0.0185) | (0.0253) |
| Condition (Control=0, Treatment=1) | 0.00139 | 0.000974 | -0.00136 | -0.0474 |
|  | (0.0202) | (0.0203) | (0.0230) | (0.0408) |
| Veracity X Condition | 0.0343*** | 0.0330*** | 0.0390*** | 0.0320 |
|  | (0.00811) | (0.00848) | (0.0105) | (0.0190) |
| Constant | 0.469*** | 0.463*** | 0.423*** | 0.440*** |
|  | (0.0177) | (0.0178) | (0.0200) | (0.0325) |
|  |  |  |  |  |
| Observations | 25,627 | 24,727 | 18,497 | 6,176 |
| Subject clusters | 855 | 825 | 617 | 206 |
| Headline clusters | 30 | 30 | 30 | 30 |
| R-squared | 0.003 | 0.004 | 0.005 | 0.006 |
| Standard errors in parentheses | | | | |
| *** p<0.001, ** p<0.01, * p<0.05 | | | | |

***Figure S1.*** *Average sharing intentions by treatment in Study 2. The 6-point Likert scale used for sharing intentions is rescaled into the interval [0,1]. This figure complements Figure 2 in the main text, which instead shows the fraction of responses above the scale midpoint. Error bars indicate 95% confidence intervals.*

***Table S6.*** *Study 2 main analysis robustness checks when excluding various headlines. Model 2 excludes headlines False-2 (which was phrased a question), False-11 (which used hedged language and so was not strictly false), and Real-9 and Real-11 (which were opinion statements). Model 3 excludes headlines False-5, False-13, and True-2, which are explicitly political in nature. Model 4 excludes those three headlines plus False-12, True-1, True-9, True-11, and True-15, which are somewhat related to politics and policy. Models 5-7 excludes headlines based on the level of partisan disagreement observed in accuracy ratings from Study 1 (defined as the absolute value of the difference between average accuracy rating given by Democrats and average accuracy rating given by Republicans).*

|  | | | (1) | (2) | (3) | (4) | (5) | (6) | (7) |
| --- | --- | --- | --- | --- | --- | --- | --- | --- | --- |
|  | | | All  headlines | Only claim  of fact | No political (narrow) | No political (broad) | Dem-Rep diff < 0.1 | Dem-Rep diff < 0.05 | Dem-Rep diff < 0.025 |
|  | | |  |  |  |  |  |  |  |
| Veracity (F=0, T=1) | | | 0.0191 | 0.0236 | 0.0190 | 0.0244 | 0.0231 | 0.0224 | 0.0368 |
|  | | | (0.0161) | (0.0155) | (0.0163) | (0.0181) | (0.0162) | (0.0197) | (0.0225) |
| Condition (Control=0, Treatment=1) | | | 0.00139 | 0.00189 | 0.00361 | 0.00396 | 0.00139 | 0.00140 | 0.00461 |
|  | | | (0.0202) | (0.0203) | (0.0202) | (0.0201) | (0.0202) | (0.0198) | (0.0201) |
| Veracity X Condition | | | 0.0343*** | 0.0322*** | 0.0305*** | 0.0306*** | 0.0327*** | 0.0304*** | 0.0299*** |
|  | | | (0.00811) | (0.00773) | (0.00781) | (0.00765) | (0.00800) | (0.00701) | (0.00350) |
| Constant | | | 0.469*** | 0.464*** | 0.473*** | 0.473*** | 0.469*** | 0.469*** | 0.469*** |
|  | | | (0.0177) | (0.0160) | (0.0179) | (0.0184) | -0.0177 | (0.0198) | (0.0159) |
|  | | |  |  |  |  |  |  |  |
| Observations | | | 25,627 | 22,208 | 23,065 | 18,794 | 24,772 | 19,646 | 11,962 |
| Subject clusters | | | 855 | 855 | 855 | 855 | 855 | 855 | 855 |
| Headline clusters | | | 30 | 26 | 27 | 22 | 29 | 23 | 14 |
| R-squared | | | 0.003 | 0.004 | 0.003 | 0.004 | 0.004 | 0.003 | 0.005 |
|  |  |  |  |  |  |  |  |  |  |
|  |  |  |  |  |  |  |  |  |  |

***Table S7.*** *Study 2 main analysis performed using multi-level model with maximal crossed random effects for subject and headline.*

Scaled residuals:

Min 1Q Median 3Q Max

-3.9851 -0.5255 -0.0066 0.5422 4.0638

Random effects:

Groups Name Variance Std.Dev. Corr

Subject (Intercept) 0.0882607 0.297087

Veracity 0.0165863 0.128788 -0.29

Headline (Intercept) 0.0018571 0.043095

Condition 0.0000858 0.009263 0.33

Residual 0.0553302 0.235224

Number of obs: 25627, groups: id, 855; item_num, 30

Fixed effects:

Estimate Std. Error df t value Pr(>|t|)

(Intercept) 4.693e-01 1.842e-02 1.689e+02 25.483 < 2e-16 ***

Veracity 1.923e-02 1.743e-02 3.676e+01 1.103 0.27714

Condition 1.184e-03 2.088e-02 8.055e+02 0.057 0.95478

Vercity:Condition 3.430e-02 1.112e-02 1.690e+02 3.085 0.00238 **

---

Signif. codes: 0 ‘***’ 0.001 ‘**’ 0.01 ‘*’ 0.05 ‘.’ 0.1 ‘ ’ 1

Correlation of Fixed Effects:

(Intr) Veracity Condition

Veracity -0.495

Condition -0.537 0.072

Veracity:Cond 0.128 -0.199 -0.326
